# PD-1/PD-L1 pathway and angiogenesis dual recognizable nanoparticles for enhancing chemotherapy of malignant cancer

**DOI:** 10.1080/10717544.2018.1509907

**Published:** 2018-11-03

**Authors:** Zhenliang Sun, Yang Zhang, Duo Cao, Xufeng Wang, Xuebing Yan, Hao Li, Linsheng Huang, Xiao Qu, Cheng Kong, Huanglong Qin, Man Wang, Wei Xu, Lin Liang

**Affiliations:** a Department of General Surgery, Shanghai Tenth People’s Hospital, Tongji University School of Medicine, Shanghai, China;; b Shanghai University of Medicine and Health Sciences Affiliated Sixth People’s Hospital South Campus, Shanghai, China;; c Department of Pharmacy, Shanghai Tenth People’s Hospital, Tongji University School of Medicine, Shanghai, China;; d The College of Life Sciences, Northwest University, Xi’an, China;; e Haiwan Community Health Center, Shanghai, China;; f Department of Orthopaedic, Tong Ren Hospital, School of Medicine, Shanghai Jiao Tong University, Shanghai, China

**Keywords:** Immunotherapy, PD-L1, tumor vasculature, dual targeting, anti-glioma

## Abstract

Although the cancer immunotherapy represents one of the most promising strategies for cancer treatment, the PD-1/PD-L1 pathway, which involves a receptor-ligand interaction, can induced immunosuppression by disabling tumor-infiltrating lymphocytes (TILs). In the present study, we coupled a PD-L1 (Programmed cell death 1 ligand 1) recognizable peptide DPPA-1 to the sequence of CGKRK, a namely tumor vasculature affinity peptide, to form a new molecule CD peptide. Thereafter, the paclitaxel (PTX)-loaded PCL nanoparticles were developed and modified with the above newly synthesized CD molecules for tumor cells and angiogenesis dual targeting drug delivery. Results of cellular experiments showed that the prepared nanoparticles have a high affinity to both tumor vasculature endothelial cells and tumor cells, which leads to an improved cytotoxicity to cancer cells and inhibition for angiogenesis. In addition, results of *in vivo* imaging assay exhibited a super tumor targeting efficacy for the CD peptide decorated nanoplatforms. Finally, the pharmacodynamic evaluation was performed and results shown that the tumor-bearing mice treated with CD-NP-PTX achieved the longest medium survival time when compared with others. Simultaneously, different nanoparticles un-loaded with drugs were also subjected to anti-tumor effect studies. Results demonstrated that the mice administrated with D-NP displayed a significantly higher ability of tumor growth inhibition when compared with the NP or C-NP, indicating a super blocking effect of PD-1/PD-L1 pathway for the ^D^PPA-1 peptide. Collectively, these results indicated that the fabricated CD-NP-PTX holds great potential in improving the tumor-targeting drug delivery efficacy and anti-glioma effect.

## Introduction

Cancer immunotherapy has attracted the most enthusiasm of many researchers for their capable of inducing durable memory responses to cope with the adaptability of tumor cells through a suit of complex mechanisms, including genomic and pathway instability (Mackensen et al., 2006; Fesnak et al., 2016). Unfortunately, the hostile tumor microenvironment can self-regulate to facilitate tumor progression by disabling tumor-infiltrating lymphocytes (TILs) which have an essential role in the activation of immune attack (Yee et al., 2002). One of the mechanisms of tumor-induced immunosuppression is the PD-1/PD-L1 pathway involves a receptor-ligand interaction (Vari et al., 2018). As the most important immunomodulatory molecules, PD-1 expression on T cells exerts an immunosuppressive effect to ‘turn off’ the cytolytic activity of TILs by interacting with PD-L1 receptors which are overexpressed on various types of cancer cells (Dömling & Holak, 2014; Armand et al., 2016; Song et al., 2018). Several lines of research works and preclinical evidence have shown that up-regulating of PD-1 significantly reduce the activity of cluster of differentiation (CD)^8+^ T cells in chronic viral infections and cancer progress (Ansell et al., 2015; Chen et al., 2017; Lavolé et al., 2018).

Because PD-1/PD-L1 pathway plays a pivotal role in immunosuppression of cancer, checkpoint receptor inhibitors are recommended as the most promising strategy for its capacity of blocking the interruption of suppressive signals in T cells from a variety of cancer (Antonia et al., 2016; Nayak et al., 2017). As researches have demonstrated previously, inhibitors such as monoclonal antibody displayed a significant effect on the eliminating of immune suppression and lead to a prolonged survival time of tumor-bearing mice (Ravi et al., 2018). Therefore, blockade of the PD-1/PD-L1 pathway is undoubtedly an effective strategy for the reversal of immune escape and immune resistance (Li et al., 2018). However, the high production cost and unavoidable immunogenicity are the most problems in applying antibody drugs for immunotherapy.

Synthetic therapeutic peptides emerged as immune drugs are proposed for many advantages, including lower costs, satisfactory stability, reduced immunogenicity, and super tumor affinity (Vlieghe et al., 2010). ^D^PPA-1 peptide is the newly discovered drug candidates developed by Chang et al. (2015). As reported, ^D^PPA-1 has high affinity to PD-L1 receptors. Moreover, in the evaluation of therapeutic efficacy of the D-peptides *in vivo*, results showed that ^D^PPA-1 significantly inhibited the progress of implanted CT26 tumor and affect negligible on the body weight change of tumor-bearing mice. Besides, the survival time of tumor-bearing mice could be prolonged to nearly 50% by treating with ^D^PPA-1. These results together suggested that application of ^D^PPA-1 treatment could be a promising strategy for cancer immunotherapy (Chang et al., 2015).

In the present study, to enhance and expand the synthetical therapy effect of cancer, we coupled the ^D^PPA-1 peptide with a namely tumor angiogenesis affinity molecule CGKRK by GYG linker (Hoffman et al., 2003). The receptors of CGKRK was heparan sulfateea sulfated polysaccharide which was highly up-regulated on tumor neovascular endothelial cells but not the normal tissues (Järvinen & Ruoslahti, 2007; Agemy et al., 2011). We named the newly synthesized sequence as CD peptide and decorated it on the surface of PTX-loaded PCL nanoparticles for tumor cells and angiogenesis dual-targeting therapy. For verification, *in vitro* cellular experiments and *in vivo* animal assay were performed.

## Materials and method

### Materials

Methoxy poly(-ethylene glycol)_3000_-poly(ε-caprolactone)_20000_ (MPEG-PCL) and maleimide-poly(ethylene glycol)_3400_-poly(ε-caprolactone)_20000_ (Mal-PEG-PCL) were obtained from Daigang Biomaterial Co., Ltd. (Jinan, China). We purchased Coumarin-6, DiR (1,1'-dioctadecyl-3,3,3',3'-tetramethyl indotricarbocyanine iodide) and 3-(4,5-dimethyl-2-thiazolyl)-2,5-diphenyl-2H-tetrazolium bromide) (MTT) from Sigma-Aldrich (St. Louis, MO). All peptides were synthesized by Bankpeptide Ltd. (Hefei, China). The 40, 6-diamidino-2-phenylindole (DAPI) was supplied by Molecular Probes, Inc. (OR, USA). Dulbecco’s modified Eagle’s medium (high glucose) (DMEM) containing 100 U/mL penicillin streptomycin, fetal bovine serum (FBS) and 0.25% trypsin-EDTA were obtained by Gibco BRL (Gaithersburg, MD). PTX and Taxol^®^ were bought from Zelang Medical Technology Co., Ltd. (Nanjing, China). All other chemicals were obtained from Sinopharm Chemical Reagent Co., Ltd. (Shanghai, China) unless mentioned otherwise.

### Cells and animals

HUVEC cells and C6 cells were obtained from the American Type Culture Collection and cultured in DMEM containing 1% penicillin/streptomycin and 10% heat-inactivated FBS. Male Sprague-Dawley rats (200 ± 20 g) and BALB/c nude mice (male, 20 ± 2 g) were provided by BK Lab Animal Ltd. (Shanghai, China) and housed under standard conditions with free access to food and water. Importantly, all of the animal experiments performed in the present study complied with the guideline of the Ethical Committee of Shanghai Jiao Tong University School of Medicine.

Tumor-bearing mice model were established as previously reported with slight modification (Ruan et al., 2017). In brief, the trypsinized C6 cells were diluted to 1 × 10^6^ cells/5 μL with PBS. Then, the mice were immobilized using a stereotactic fixation device and anesthetized with 5% chloral hydrate. Subsequently, 5 μL of cell suspension was slowly injected into the right corpus striatum of nude mice. After the wound being stitched, the tumor-bearing mice were raised under standard condition.

## Methods

### Preparation of peptide-modified nanoparticles

The blend of MPEG-PCL (18 mg) and Mal-PEG-PCL (2 mg) were dissolved by 1.0 mL of dichloromethane. Then, 2.0 mL of 1% sodium cholate solution was added into the mixture, followed by rapidly stirring for 5 min. Subsequently, a probe sonicator (Ningbo Scientz Biotechnology Co. Ltd., China) was introduced to fabricate the nanoparticles (NP) through ultrasonication for 3.0 min at 320 w. After the resulting O/W emulsion was diluted with 5 mL of 0.5% sodium cholate solution, the residual dichloromethane in the mixture solution was removed using a ZXB98 rotavapor (Shanghai Institute of Organic Chemistry, China). Finally, the nanoparticles were collected by centrifuging at 14,000 rpm for 45 min under 4 °C. To decorate on the surface of nanoparticles with peptide, the obtained NP was resuspended with distilled water and poured into a container. Thereafter, CD peptide was added and allowed to react with the Mal- under room temperature. Importantly, the molar ratio of CD peptide to Mal-PEG-PCL was 1.5:1. After 6 h of incubation, the nanoparticle suspension was subjected to centrifuging at 14,000 rpm for 45 min to remove the unconjugated peptide. Finally, the obtained peptide modified nanoparticles (CD-NP) were lyophilizated and preserved at 4 °C for further use. Of great importance, to minimize the influence of functionalization, the unmodified nanoparticles were treated in the same way as that for the preparation of CD-NP but without adding peptides for conjugation. The drug-loaded nanoparticles (NP-PTX) and Coumarin-6- or DiR-labeled nanoparticles (NP-C6 or NP-DiR) were developed with the same method except for the addition of 0.2 mg of PTX, Coumarin-6, and DiR, respectively.

### Characterization of NP-PTX and CD-NP-PTX

The morphologies of the prepared nanoparticles were determined using the transmission electron microscope (TEM, JEM-1200EX, JEOL, Tokyo, Japan) after the nanoparticles were negatively stained with sodium phosphortungstate solution and placed on a carbon film-coated copper grid. The particle sizes, polydispersity indexs (PDIs), and zeta potentials of NP-PTX and CD-NP-PTX were evaluated using the dynamic light scattering (DLS, Zetasizer Nano-ZS, Malvern, UK). The drug-loading content (DL) and drug encapsulation efficacy (EE) in different nanoparticles were further studied. For experiments, 20 µL prepared nanoparticle solutions were suspended by distilled water and added into 80 µL acetonitrile to disrupt the core-shell structure of nanoparticles. Thereafter, the PTX concentration was examined using the high-performance liquid (HPLC) system with the determine wavelength was set at 227 nm. Finally, the DL and EE were calculated according to the following formulas (the investigation was replicated three times):

$$DL\left(\%\right)=\frac{\text{The amount of drug in nanoparticles}}{\text{Nanoparticle weight}}\;\times \;100\%$$

and

$$EE\left(\%\right)=\frac{\text{The amount of drug in nanoparticles}}{\text{Toal amount of drug added}}\;\times \;100\%.$$

We further investigate the critical micelle concentration (CMC) of the developed nanoparticles using pyrene as the fluorescence probe and determined the result *via* a fluorescence spectrophotometer as previously reported (Huang et al., 2014). Moreover, the stability of nanoparticle formulations including NP-PTX and CD-NP-PTX were further investigated with the DMEM containing 20% FBS acting as the media. Then, the sizes of different nanoparticles were measured carefully at predetermined time points.

### *Drug release profiles of nanoparticles* in vitro

Drug release profiles of nanoparticles were investigated through an equilibrium dialysis method and with the PBS (pH 7.4) containing 10% rat plasma acting as the release medium. For experiments, lyophilized NP-PTX and CD-NP-PTX were suspended, respectively, and the PTX concentration was diluted to 100 µg/mL by the release medium. Subsequently, the prepared samples were added into a dialysis bag (MWCO =8000 Da, Greenbird Inc., Shanghai, China) and immersed into a tube containing 30 mL release medium. After that, the tubes were immediately placed at a table concentrator under the condition of 37 °C and 100 rpm. For drug release detection, 0.2 mL of the medium was obtained and replenished with equal volume of fresh release medium at predetermined time points (0.5, 1, 2, 3, 4, 6, 8, 10, 12, 24, 36, and 48 h). The concentrations of PTX were analyzed using HPLC with the wavelength was set at 227 nm. More importantly, to verify the coumarine-6 can be used as the fluorescence probe for cellular or *in vivo* investigation, the release behavior of coumarine-6 from nanoparticles were also studied as above.

### Cellular uptake experiments

HUVEC cells and C6 cells were seeded in the 12-well plate, respectively, at the density of 5 × 10^3^ cells/cm^2^. Twenty-four hours later, both cells were treated with various coumrin-6-loaded nanoparticles with nanoparticles concentration ranging from 10 to 200 μg/mL. After 1 h of incubation, the cells were rinsed by cold PBS and subsequently fixed with 4% formaldehyde for 10 min. For qualitative evaluation, the fluorescent intensity of cells treated by different nanoparticles was observed under a fluorescence microscope (Olymus IX71, Japan).

To quantitative study, the cellular association of different nanoparticles, both HUVEC cells and C6 cells were seeded in 12-well plates at the density of 5 × 10^4^ cells per well. Post-treating with various coumarin-6 labeled-nanoparticles, both cells were washed twice with PBS and collected by centrifugation at 1000 rpm for 5 min after 1 min of trypsinization. Then, the fluorescence intensity of each group was determined using an FACS Aria Cell Sorter (BD, USA).

### Tumor cell proliferation assay

C6 cells were seeded in the 96-well plates at a density of 5 × 10^3^ cells/well and exposed to different PTX formulations after 24 h of incubation. The PTX formulations are including Taxol^®^, NP-PTX, C-NP-PTX, and CD-NP-PTX and the concentration of drug are ranging from 0.001 to 1 μg/mL. After incubation of cells with various PTX formulations for 48 h, 20 μL of MTT solution (5 mg/mL) was added into each well of plates and allowed to interact with living cell for 4 h. Thereafter, each well of the plates was supplemented with 150 μL of DMSO followed by gently shaken it for 10 min to completely dissolve the formed formazan. Then, the cell viability was analyzed using a microplate reader (Thermo Multiskan MK3, USA) with the excitation wavelength was set at 570 nm. Importantly, cells treated with DMEM alone were set as control.

### Angiogenesis inhibition experiments

After pre-chilling the 96-well plates for 12 h under the condition of −20 °C, 100 μL of growth factor-reduced Matrigel was coated on the bottom of each well. Then, the plates were incubated at 37 °C for 45 min to polymerize the Matrigel. To form the tumor angiogenesis, 1 × 10^4^ HUVEC cells, which have been diluted with DMEM containing various PTX formulations (Taxol^®^, NP-PTX, C-NP-PTX, D-NP-PTX, and CD-NP-PTX), were seeded onto the Matrigel followed by being gently shaken for 1 min. The PTX concentrations were set at 1 nM, 5 nM, and 10 nM, respectively, and cells without any treatment were acted as the control group. After 10 h incubation, the branches of capillary-like tube were observed under a phase-contrast microscopy. Concomitantly, statistical analysis (*n =* 3) was also conducted for quantitative evaluation.

### In vivo *imaging analysis*

To evaluate the bio-distribution of nanoparticles, six tumor bearing-mice were randomly divided into two groups (*n =* 3) and injected with DiR-labeled NP and CD-NP, respectively. The dosage of DiR was set at 5 mg/kg. Then, a real-time imaging was subsequently performed using the *in vivo* imaging system (PerkinElmer, Maestro, Waltham, MA). Twenty-four hours after the injection, all of the mice were sacrificed with the main organs (including heart, liver, spleen, lung, kidney, and glioma-bearing brain) were obtained. After washed twice with saline, qualitative and semi-quantitative analysis of nanoparticles distribution in tissues were conducted, respectively, through the CRi *in vitro* imaging system.

### Biodistribution of nanoparticles

To investigate the distribution of nanoparticles in tumor site, six glioma-bearing mice were randomly grouped (*n =* 3) and administrated with NP or CD-NP *via* the tail veil. Coumarin-6 was used as the fluorescence probe to visualize nanoparticles and its dosage was set at 1 mg/kg. Six-hours later, all of the mice were anesthetized with 5% chloral hydrate followed by perfusion with saline and 4% paraformaldehyde. Then, the glioma-bearing brains were collected and embedded in 4% paraformaldehyde. After fixing for 24 h, the brains were subjected to sectioning for 14 µm slides, followed by staining with DAPI. Finally, the distribution of nanoparticles in tumor tissues was observed under a confocal microscopy analysis system (LSM710, Leica, Germany).

### Pharmacokinetics study

To determine the drug blood retention effect of these developed nanoparticles, the plasma pharmacokinetics (PK) of PTX was examined. Briefly, 15 male SD rats (200 ± 20 g) were randomly divided into five groups (*n =* 3) and intravenously administered with Taxol, NP-PTX, C-NP-PTX, D-NP-PTX, and CD-NP-PTX, respectively, *via* the tail vein at an equivalent PTX dosage of 5 mg/kg. After that, the blood samples were drawn from the retinal vein plexus at predetermined time points of 0.083, 0.25, 0.5, 1, 2, 3, 4, 6, 8, 12, and 24 h post-injection and centrifuged immediately at 3000 rpm for 10 min. To analyze the collected samples, 150 μL of methanol was added followed by centrifuging at 12,000 rpm for 10 min to extract the PTX. Then, an equal volume of deionized water was added into the supernatant followed by vortexing for 5 min. Finally, the pharmacokinetic data analysis was performed *via* a liquid chromatography-tandem mass spectrometry (LCMS/MS) analysis.

### Anti-tumor effect

To evaluate the anti-tumor effect of various PTX formulations, 24 glioma-bearing mice were randomly grouped (*n =* 6) and intravenously injected with PBS, Taxol^®^, NP-PTX, C-NP-PTX, D-NP-PTX, and CD-NP-PTX, respectively. The PTX dosage was set at 5 mg/kg and the treatment was done every other day in a week. Then, the survival times of each mouse were carefully recorded. Furthermore, to evaluate the role of PD-1/PD-L1 pathway in tumor growth and progress, the randomly grouped (*n = *6) glioma-bearing mice were treated by NP, C-NP, D-NP, and CD-NP, respectively. Then, the survival times of each mouse were also evaluated in the following days.

### Statistical analysis

Results are presented as mean ± standard deviation (SD). Statistical evaluation was conducted using one-way ANOVA, followed by Bonferroni post hoc test for multigroup comparison. Statistical significance was defined as *p* < .05.

## Results

### Characterization of NP-PTX and CD-NP-PTX

**Figure 1.**
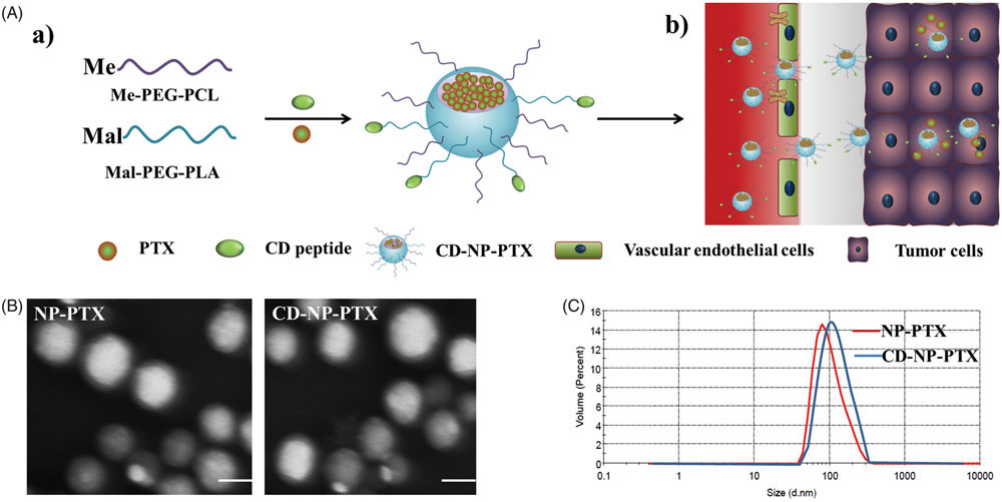
Preparation and characterization of nanoparticles. (A) Scheme of fabrication of CD-NP-PTX (a) and targeting delivery of drugs to tumor site (b). (B) TEM images of different nanoparticles. (C) Size distribution of nanoparticles determined by the DLS analysis. Bar =100 nm.

Morphological characteristics of NP-PTX and CD-NP-PTX obtained using the TEM showed that the prepared drug-loaded nanoparticles have an uniform spherical morphology (Figure 1(B)). Results of DLS analysis presented that the average dynamic diameter of NP-PTX was about 96.21 ± 3.21 nm, and the nanoparticles have a narrow size distribution with the PDI was only 0.119 ± 0.127 (Figure 1(C)). In addition, the zeta potential of NP-PTX was also examined and showed a negative charge of −28.36 ± 2.54 mV. Of great importance, after the modification of CD peptide, the physicochemical properties of nanoparticles did not change obviously with the particles size, PDI, and zeta potential of CD-NP-PTX were 103.57 ± 3.57 nm, 0.134 ± 0.134, and −26.33 ± 3.29 mV, respectively.

**Figure 2.**
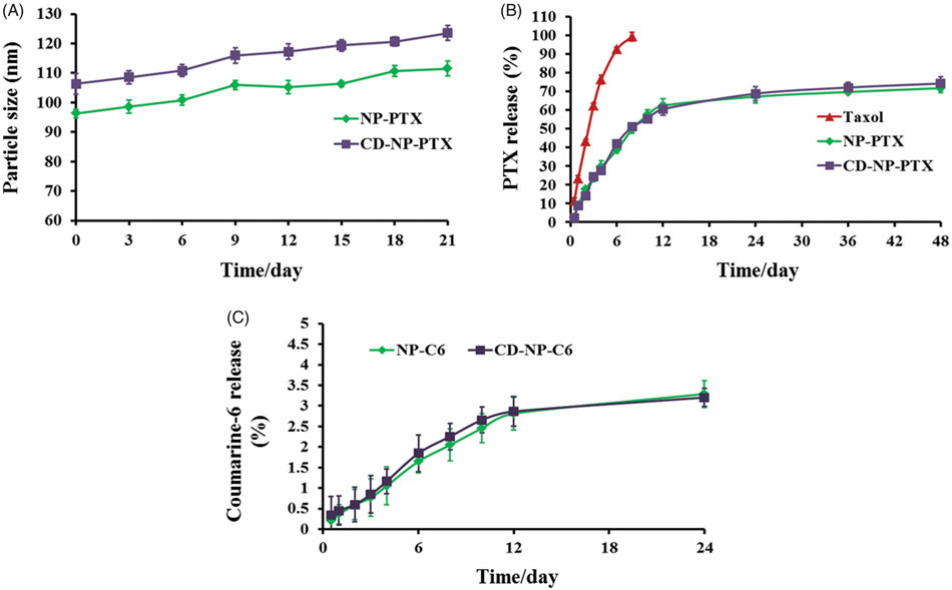
(A) Stability investigation of nanoparticles *in vitro* with the DMEM containing 20% FBS acting as the medium. (B) Drug release profiles of NP-PTX and CD-NP-PTX with the Taxol^®^ as the control group. (C) *In vitro* investigation of the coumarine-6 release behavior from NP-C6 and CD-NP-C6.

As calculated using the drug-loading content and drug encapsulation efficacy equation, the DL and EE of NP-PTX were1.6 ± 1.17% and 57.21 ± 2.42%, respectively. For the investigation of CD-NP-PTX, the DL and EE were 1.47 ± 1.34% and 55.23 ± 3.07%, respectively. The results of CMC investigation exhibited that both of nanoparticles displayed a similar critical micelle concentration with the values were 0.291 mg/L for NP-PTX and 0.314 mg/L for CD-NP-PTX, respectively. For stability investigation, the prepared drug delivery system displayed a negligible change of particle size throughout the total experimental times when incubated in PBS containing 20% FBS (Figure 2(A)), indicating a satisfactory stability for CD-NP-PTX.

### *Drug release profile of nanoparticles* in vitro

The drug release profiles of nanoparticles were studied *in vitro* and compared with the Taxol^®^. As demonstrated in Figure 2(B), both NP-PTX and CD-NP-PTX displayed a controlled drug release behavior and similar cumulative PTX release at all the time points. After 48 h of incubation, 69.31% of PTX in NP-PTX and 72.16% in CD-NP-PTX were released, respectively. However, in the case of Taxol^®^, a burst drug release was observed as the cumulative PTX release at 8 h was over 98%. For the investigation of coumarine-6 release, results showed that almost all the coumarin-6 remained in NP-C6 and CD-NP-C6 during the experimental period with the cumulative coumarine-6 release at 24 h were both below 4%, indicating that the fluorescent signal of coumarin-6 could represent the fate of nanoparticles both *in vitro* and *in vivo* (Figure 2(C)).

### Cellular uptake experiments

**Figure 3.**
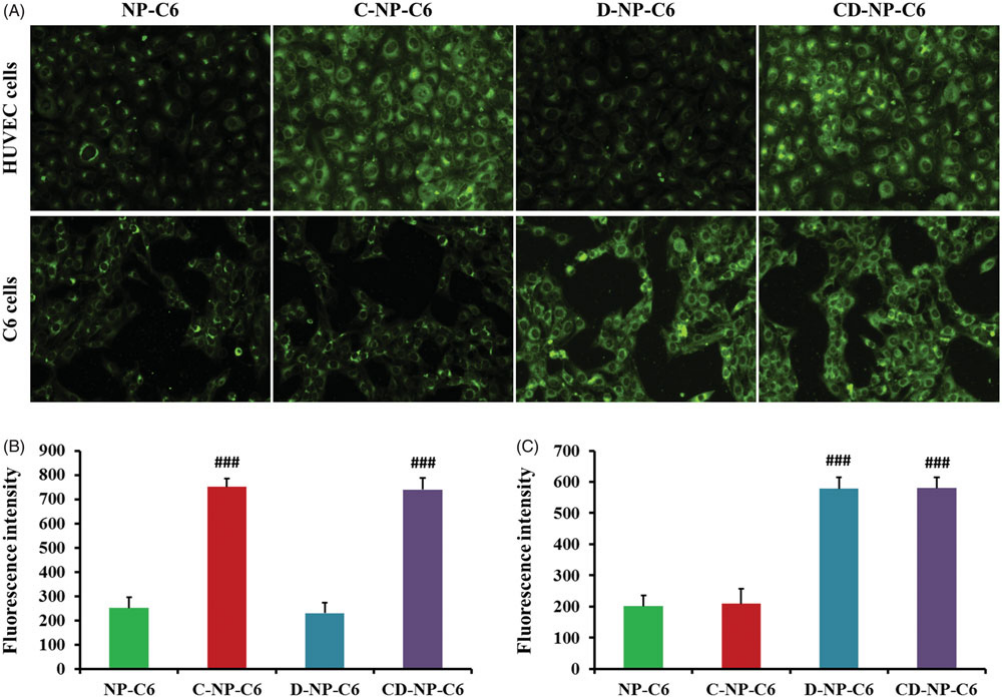
Cellular association of nanoparticles *in vitro* with the coumarin-6 acting as the fluorescence probe. (A) Qualitative images of cells obtained by the fluorescence microscope after incubated cells with different nanoparticles. (B) Quantitative analysis of HUVEC cellular uptake of various nanoparticles post 1 h of incubation. (C) Quantitative analysis of C6 cellular uptake of various nanoparticles post 1 h of incubation. ###*p* < .001 significantly higher than the cellular uptake of unmodified NP-C6.

Cellular uptake assay was performed using the qualitative and quantitative analysis, respectively, with coumarin-6 acting as the fluorescence probe. As results showed in Figure 3(A), HUVEC cells treated with NP-C6 exhibited the weakest fluorescence intensity as compared with other groups. However, the green signal in cells increased obviously (3.02 folds) after modified the nanoparticles with C peptide, indicating that the C-NP-C6 have a high affinity to HUVEC cells. What’s more, cells incubated with D-NP-C6 displayed similar fluorescence intensity to the group treated by NP-C6, suggesting that HUVEC cells are negative to the expression of PDL-1. In this case, cellular association of CD-NP-C6 and C-NP-C6 showed negligible difference as observed using the fluorescence microscope. For the PDL-1 positive C6 cells, D and CD peptide decorated nanoparticles exhibited the highest fluorescence intensity, whereas a similar green signal was observed in NP-C6 and C-NP-C6 treated cells. Quantitative analysis further confirmed the above results (Figure 3(B,C)).

**Figure 4.**
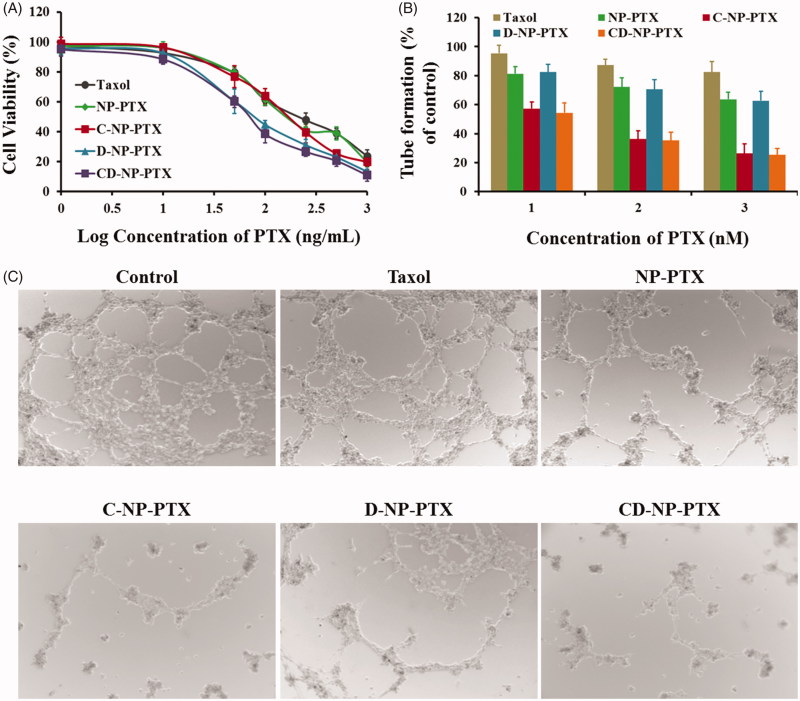
(A) Cytotoxicity of Taxol^®^, NP-PTX, C-NP-PTX, D-NP-PTX, and CD-NP-PTX against C6 cells *in vitro* after 48 h of incubation. Investigate the angiogenesis inhibition of different PTX formulations by the tube formation method. Quantitative (B) and qualitative (C) analysis of tube networks after treated with Taxol^®^, NP-PTX, C-NP-PTX, D-NP-PTX, and CD-NP-PTX, respectively, at the PTX concentration of 10 nM. The drug-free DMEM treated group was served as the control.

### Tumor cell proliferation assay

Cytotoxicity of various PTX formulations against tumor cells was investigated using the MTT method. Results shown in Figure 4A exhibited that all the PTX formulations inhibited tumor cell proliferation in a dose-dependent manner. Consistent with the cellular uptake results, cells treated with D-NP-PTX and CD-NP-PTX displayed the lowest cell viability compared with other groups with the IC_50_ value were 66.84 ng/mL and 61.27 ng/mL, respectively. Besides, the IC_50_ value of NP-PTX and C-NP-PTX were 138.3 ng/mL and 129.7 ng/mL, respectively, which are both significantly lower than that of Taxol^®^ (191.8 ng/mL).

### Angiogenesis inhibition experiments

The effect of PTX formulations on the formation of angiogenesis was evaluated in this study. As shown in Figure 4(B,C), the drug-free DMEM treated group exhibited the most extensive and enclosed tube networks compared with others. After exposed to Taxol^®^, a negligible tube formation inhibition was observed. However, a less channel destruction could be obtained by incubating cells with NP-PTX. After decoration of nanoparticles with CGKRK peptides, the angiogenesis was significantly inhibited, indicating a highly angiogenesis homing property of this peptide. In contrast, there was no obvious difference between the group treated by NP-PTX and D-NP-PTX, which was mainly because the fact of ^D^PPA-1 is just a tumor cell recognizable peptide. In this case, the C-NP-PTX and CD-NP-PTX treated group displayed the similar activity in anti-angiogenesis formation.

**Figure 5.**
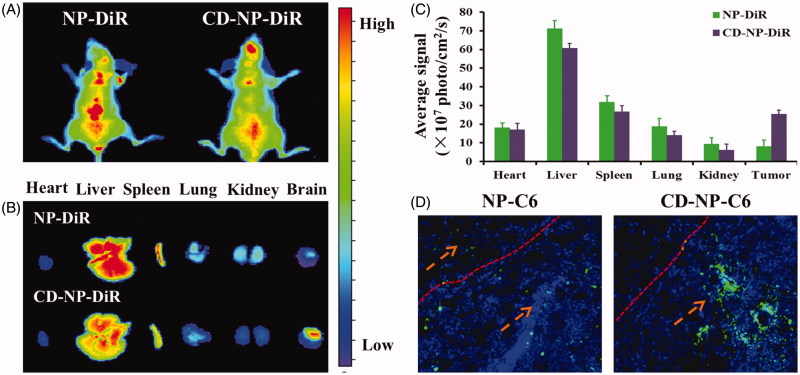
(A) *In vivo* biodistribution of DiR-labeled nanoparticles in glioma-bearing mice. (B) Ex vivo optical images of dissected tumors and main organs at 24 h post injection. (C) Semiquantitative analyzing the distribution of nanoparticles in various organs and tumors. (D) Evaluation of tumor targeting effect by study the distribution of nanoparticles in tumor site. Blue are the DAPI stained cell nuclei and green represented the coumarin-6-labeled nanoparticles.

### In vivo *imaging analysis*

The noninvasive NIR fluorescence imaging was conducted to study the targeting effect of nanoparticles in tumor-bearing mice. As results are shown in Figure 5(A), mice treated with NP-DiR displayed a weak fluorescence signal at tumor site, indicating a limited tumor accumulation of NP-DiR. In comparison, more selective accumulation of CD-NP-DiR at tumor site could be seen at 24 h post injection, suggesting that CD peptide functionalized nanoparticles are highly affinity to glioma. Moreover, further *ex vivo* imaging and semiquantitative fluorescence analysis showed that modification of CD peptide could also down-regulated the accumulation of nanoparticles in normal tissues (Figure 5(B,C)).

**Figure 6.**
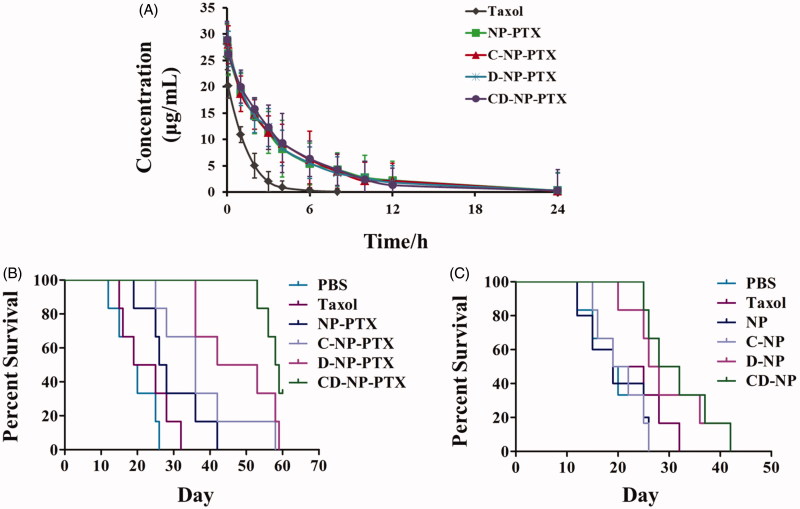
(A) Pharmacokinetic study of different PTX formulations *in vivo*. (B) Kaplane Meier survival curve of mice bearing glioma treated with PBS, Taxol^®^, NP-PTX and C-NP-PTX, D-NP-PTX, and CD-NP-PTX, respectively at PTX dose of 5 mg/kg (*n =* 6). (C) Evaluation of immunotherapy effect of ^D^PPA-1 peptide by investigating the survival time of glioma-bearing mice post treat with different nanoparticles (*n =* 6).

### Biodistribution of nanoparticles

As clearly illustrated in Figure 5(D), more accumulation of nanoparticles at tumor tissue could be achieved by decorating the drug delivery system with CD peptide. Besides, the unmodified nanoparticles not only accumulated in tumor sites but also the normal brain tissues, which will lead to severe side effect.

### Pharmacokinetics study

As shown in the plasma concentration-time curves (Figure 6(A)), Taxol^®^ exhibited the rapidest clearance when compared with other formulations with the PTX concentration was undetectable at 12 h. In comparison, the PTX-loaded nanoparticles displayed a higher concentration at all the time points, indicating that the prepared nanoparticles could significantly prolonged the elimination half-life and decreased clearance rate. More importantly, the similar pharmacokinetic curve between NP-PTX and various peptides functionalized ones suggested that modification of peptide did not affect pharmacokinetic properties of NP-PTX. Pharmacokinetic parameters were further calculated and results shown that an obviously prolonged half-life-time (*t*_1/2_) was achieved by those developed nanoparticles when compared with Taxol^®^. The half-life-time of each formulation was 6.1 h for NP-PTX, 6.9 h for C-NP-PTX, 6.6 h for D-NP-PTX, and 7.2 h for CD-NP-PTX, whereas that of Taxol^®^ was 3.2 h. Moreover, the area under the curve (AUC) of CD-NP-PTX was more 13-fold higher than that of Taxol, whereas similar to that of C-N-PPTX, D-NP-PTX, and NP-PTX.

### Anti-tumor effect

As results shown in Figure 6(B), the mice given with CD-NP-PTX achieved the best treatment effect with a medium survival time of 58.5 days, while the mice treated by PBS, Taxol^®^, NP-PTX, C-NP-PTX, and D-NP-PTX achieved the medium survival time was 19.5, 22, 27, 36, and 47.5 days, respectively. For the evaluation immunotherapy of ^D^PPA-1 peptide, the survival time of mice treated with PTX free nanoparticles was also studied. As illustrated in Figure 6(C), mice treated with D-NP and CD-NP exhibited the lowest mortality compared with others, whereas there is no significant difference among the mice treated by PBS. The median survival time of each group was 19 (NP), 20.5 (C-NP), 27 (D-NP), and 30 (CD-NP) days, respectively.

## Discussion

Paclitaxel (PTX) is a widely used chemotherapeutic agent and showed anti-neoplasic activity against various types of solid tumors such as ovarian, breast, and lung cancers and has also been proven effective in the treatment of gliomas (Hájek et al., 1996; Zhan et al., 2010). However, the chemotherapy of tumors is always severely discounted by the immunosuppression of tumor cell through a complicated process (Dudley et al., 2001). Blockade of the protein–protein interaction between the transmembrane protein programed cell death protein 1 and its ligand PD-L1 has emerged as a promising immunotherapy for treating cancers (Prodeus et al., 2015). In this study, we initiated a novel combination of immunotherapy and chemotherapy for cancer by decorating a chemotherapeutics-loaded nanoparticle with PD-1/PD-L1 antagonist-^D^PPA-1 peptide and tumor angiogenesis targeting peptide CGKRK. We choose the nanoparticles as the platform in the present work is mainly depends on the ratio of molecular weight between PEG and PLA (Xia et al., 2011; Zhao et al., 2012).

As demonstrated using the TEM images and DLS analysis, the modification of peptide have negligible effect on the physicochemical properties of the prepared nanoparticles. Besides, drug release investigation showed that NP-PTX and CD-NP-PTX have a similar controlled drug release behavior with a cumulative PTX release at 48 h were 74.23% and 76.27%, respectively. For cancer-targeting therapy, a stable drug delivery system is the most prerequisite for precisely delivering therapy agents to tumor site. As demonstrated clearly, the nanoparticles developed in this study did not show obviously particle size change when incubated in the medium of DMEM supplemented with 20% FBS, indicating a satisfactory stable of NP-PTX and CD-NP-PTX.

As the classical model of mimic tumor neovascular cells, HUVEC cells are reported with overexpression of p32 receptors. In this case, we modified the nanoparticles with CGKRK peptide, a namely p32 recognizable molecule, for anti-angiogenesis therapy. For the verification, cellular uptake assay was performed. As demonstrated in Figure 3, C-NP-C6 treated cells exhibited a stronger fluorescence signal compared with NP-C6. Besides, no significant difference between cellular association of NP-C6 and D-NP-C6 suggested that HUVEC are positive for the expression of p32 but not the PDL-1. Furthermore, we also performed the angiogenesis inhibition experiments by tube formation method. As results demonstrated that cell treated with C-NP-PTX and CD-NP-PTX exhibited the highest degree of tube damage compared with other groups. These results together suggesting that the C peptide modified nanoparticles are an efficient angiogenesis inhibitor.

As demonstrated previously, PD-L1 is highly up-regulated on various types of cancer cells (Chang et al., 2015), and thus it can be used as the ideal target for targeting tumor drug delivery. ^D^PPA-1 peptide, a namely antagonists of PD-1/PD-L1 pathway, was reported highly specific to PD-L1 receptors. Based on that, we modified on the PTX-loaded nanoparticles with ^D^PPA-1 peptide for tumor cells targeting therapy. As demonstrated using the qualitative and quantitative analysis of cellular uptake, C6 cells treated with D-NP-C6 and CD-NP-C6 displayed the strongest fluorescence intensity. Moreover, consistent with cellular uptake results, D-NP-PTX and CD-NP-PTX treated cells exhibited the lowest cell viability compared with others (Figure 4). These results together confirmed that the ^D^PPA-1 peptide functionalized nanoparticles have high affinity to tumor cells.

*In vivo* targeting experiments were further performed, as results showed in Figure 6 displayed that CD peptide decorated nanoparticles exhibited the more tumor accumulation compared with the unmodified ones. Bio-distribution of nanoparticles showed that CD-NP-C6 treated glioma-bearing mice displayed stronger fluorescence signal than the undecorated NP-C6. More importantly, the CD peptide functionalized nanoparticles exhibited negligible distribution in normal brain tissue, whereas the NP-C6 showed an obviously normal tissue penetration.

Finally, we investigated the anti-tumor effect of various PTX formulations by study the median survival time of glioma-bearing mice. Results showed in Figure 6(B) illustrated that the mice treated with CD-NP-PTX achieved the longest median survival time compared with other groups. Moreover, as demonstrated previously, ^D^PPA-1 peptide could be a promising drug candidate for cancer immunotherapy through blockading of PD-1/PD-L1 pathway. In this case, we have studied the therapy effect of ^D^PPA-1 peptide on glioma. As confirmed by the survival results (Figure 6(C)), mice treated with D-NP and CD-NP exhibited the lowest mortality compared with others, suggesting an immunotherapy effect of ^D^PPA-1 peptide. In comparison, there is no significant difference among the mice treated by PBS, NP, and C-NP, indicating that blank NP and C peptide affect negligible on glioma.

## Conclusion

In summary, we designed and prepared a peptide-mediated nano-DDS by conjugating peptides ^D^PPA-1 and CGKRK to the surface of PEG-PCL nanoparticles *via* a maleimide–thiol reaction. ^D^PPA-1, a PD-L1 affinity peptide, could reverse the immune escape and suppression of tumor cells by blockading the PD-1/PD-L1 pathway. CGKRK, a tumor vasculature homing peptide, was widely used for anti-angiogenesis therapy. In this case, the developed CD-NP is expected to have improved glioma targeting ability and enhanced chemotherapy by introducing immunotherapy. PTX-loaded CD-NP was spherical with a mean particle size of 103.57 nm and a zeta potential of −26.33 mV. The release of PTX from PC-NP-PTX was confirmed *in vitro*. The modification of PEG-PCL nanoparticles with CD peptide significantly increased the cytotoxicity of its payload PTX against C6 cell. *In vivo* fluorescence imaging confirmed that CD-NP could accumulate effectively and penetrate deeply in GBM tissue. *In vivo* anti-GBM evaluation revealed that CD-NP-PTX exhibited powerful anti-GBM efficacy with the longest survival time among all the treatments. Taken together, our findings indicated CD-NP as a promising nano-DDS for glioma targeting delivery of anticancer drugs.
